# To what degree does hybridization facilitate evolutionary radiations in mountain areas?

**DOI:** 10.1093/nsr/nwac288

**Published:** 2022-12-19

**Authors:** Siri Birkeland

**Affiliations:** Faculty of Chemistry, Biotechnology and Food Science, Norwegian University of Life Sciences, Norway; Natural History Museum, University of Oslo, Norway

Mountains are sometimes referred to as ‘sky islands’ since their biodiversity may be geographically isolated at high altitudes in a sea of uninhabitable lowlands (first used by [1]). It is a nice expression because it gives an immediate picture of how mountain systems may behave like oceanic islands, especially when it comes to patterns of dispersal and colonization. Sky islands are also known for evolutionary radiations just like other island systems. New ecological opportunities in connection with mountain uplift are assumed to trigger accelerated diversification, and mountain radiations are generally both young and fast [2]. Ma *et al*. [3] study such a young mountain radiation in south-west China, an area which includes some of the most biologically diverse mountain ranges in the world. They show that hybridization may have been common throughout the radiation of Rhododendron subgenus *Hymenanthes*, even though species are spread over isolated sky islands. Hybridization has fuelled some of the most iconic radiations in other island-like systems by increasing genetic diversity and morphological variation (e.g. Darwin's finches in the Galapagos Islands, and the Cichlid fishes in the African Great Lakes; [4,5]). However, in the case of Rhododendron it is still unclear whether hybridization has driven speciation, which is also pointed out by the authors. What is clear is that the members of *Hymenanthes* have not been entirely isolated at their sky islands throughout the radiation, and that hybridization has been common. This indicates that speciation with gene flow may have taken place.

There are at least two different possibilities that could explain the lack of isolation and facilitate potential speciation with gene flow. First, there might have been continuous gene flow between sky islands, but at such low levels that divergence could still occur (i.e. parapatric speciation). Rhododendron species are good seed dispersers and show large variation in pollinators including birds, so gene flow between sky islands could happen due to long-distance dispersal of seeds and pollen. In addition, one could imagine that the many diverse pollinators may have provided new avenues for non-random mating. Second, there may have been periods of both isolation and gene flow consistent with a mixing-isolation-mixing (MIM) model (as described by [6]). Since not all mountain species are equally good dispersers or even possess the same type of pollination biology, a MIM scenario is perhaps a more interesting possibility as it can represent a general model for how radiations occur in mountains. The MIM model suggests that speciation is driven by alternating periods of isolation and gene flow. Periods of isolation may allow time for divergence, while periods of gene flow may fuel speciation by, for instance, increasing genetic diversity. According to He *et al*. [6], such cycles may lead to higher diversification rates than allopatric models, which could help explain rapid radiations. In mountain systems, it is assumed that climatic fluctuations may cause such MIM scenarios, and there even exists a mountain-specific equivalent called ‘flickering connectivity system’ (FCS; [7]). The FCS is a framework for understanding exactly how cyclical phases of connectivity and isolation, driven by Pleistocene climate change, may have led to increased diversification in combination with mountain topography. The logic behind FCS is that climate cooling may cause distribution areas to move downwards and create corridors of connectivity between previously isolated populations in the lowlands, while climate warming may cause distribution areas to move upwards and become fragmented. The main point of the FCS is that cycles of isolation and contact may promote speciation, but unlike MIM it also puts weight on mountain specific variables like topography and Pleistocene climate. Taken together, a MIM or FCS scenario is plausible in mountain radiations, but a challenge remains to link periods of gene flow to periods of connectivity by, for instance, including geological data on barriers. Further work on the role of hybridization in mountain radiations can help reveal the extent to which hybridization may have contributed to rapid speciation in this and other mountain radiations.
